# Cerebral Venous Sinus Thrombosis Triggered by Severe Dehydration

**DOI:** 10.7759/cureus.74654

**Published:** 2024-11-28

**Authors:** Shruti Wadhwani, Nikita Wadhwani, Sameh Elias

**Affiliations:** 1 Internal Medicine, Hackensack Meridian Health Palisades Medical Center, North Bergen, USA

**Keywords:** cerebral venous sinus thrombosis (cvst), dural venous sinus thrombosis, headaches, intracranial hemorrhage, thrombophilias

## Abstract

Cerebral venous sinus thrombosis (CVST) is the causative factor in a small proportion of strokes. It primarily affects individuals aged less than 55 years, with up to two-thirds of cases affecting females. It can be precipitated by a myriad of transient or permanent risk factors that result in a prothrombotic state. Diagnosis of CVST requires a high index of clinical suspicion as the presenting symptoms are often vague and include headaches, visual deficits, seizures, etc. Computed tomography or magnetic resonance venography are sensitive imaging diagnostic modalities. The majority of patients have a favorable prognosis. It is important to pursue thrombophilia work-up after the resolution of an acute episode as some cases are secondary to an underlying malignancy.

## Introduction

Headache is the most common presenting symptom in cerebral venous sinus thrombosis (CVST), occurring in almost 90% of cases. Symptoms have an insidious onset, with most patients presenting after 24-48 hours after onset. This happens due to an increase in intracranial pressure or juxtacortical parenchymal injury. Seizures and/or focal neurological deficits are seen in 20-50% of cases [1]. Visual disturbances (diplopia, transient visual obscuration) can affect up to one-third patients. Cranial neuropathies affect about 10% of patients. Incidence of encephalopathy and coma has been reported to be as high as 20% in some studies [2]. 

Risk factors for CVST could either be transient or chronic in nature. Oral contraception and hormone replacement therapy greatly increase the risk of CVST in young women. Pregnancy, obesity, dehydration, COVID-19 infection, head and neck infections, head injury, lumbar puncture, neurosurgical procedures are other transient risk factors associated with CVST. Chronic factors include hereditary (protein C or S deficiency, factor V Leiden, prothrombin G20210A mutation), and acquired thrombophilias (antiphospholipid antibody syndrome, Janus kinase 2 (JAK2) mutation, malignancy, inflammatory bowel disease, Behçet disease, etc.) [3].

Herein we present a case of CSVT that was precipitated by marked dehydration. Prompt treatment with anticoagulation resulted in complete neurological recovery. Requirement for informed consent was waived by the IRB, and all patient data has been de-identified. 

## Case presentation

A 59-year-old female with no past medical history presented with three days of headache and nausea that began after she worked in the yard all morning in high heat without having eaten anything, and with very little hydration. She then had a near-syncopal event that was witnessed by her son. Symptoms improved minimally after the initial event. She denied recent trauma, fevers, chills, upper respiratory infections, skin lesions, or any abdominal or urinary complaints. 

Family history was non-contributory. The patient was not taking any medications. She was up-to-date with her screening procedures and vaccinations. Vitals were stable on presentation except for a blood pressure of 92/56 mmHg. The National Institutes of Health Stroke Scale (NIHSS) score was 0. Neurological examination did not reveal any abnormalities. The patient was alert and oriented to time, place, and person. Cranial nerve examination was normal. No visual field defects were evident. Facial strength and sensation were intact. Motor examination revealed normal muscle bulk and tone. Strength was 5/5 across all extremities. Sensation to light touch and pinprick was intact. Coordination testing disclosed no abnormalities. 

**Figure 1:**
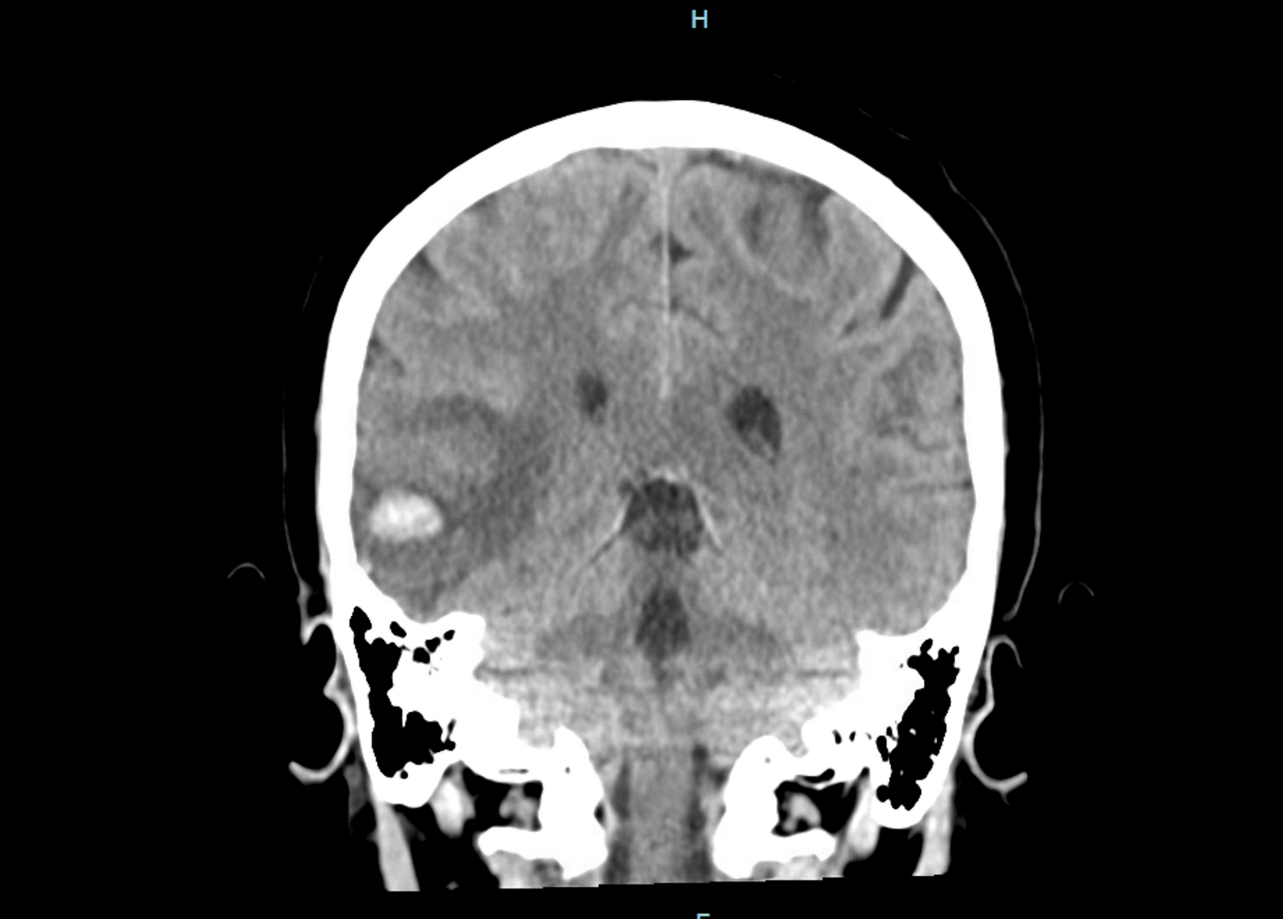
CT head without contrast showing acute right temporal intraparenchymal hematoma (coronal view)

**Figure 2:**
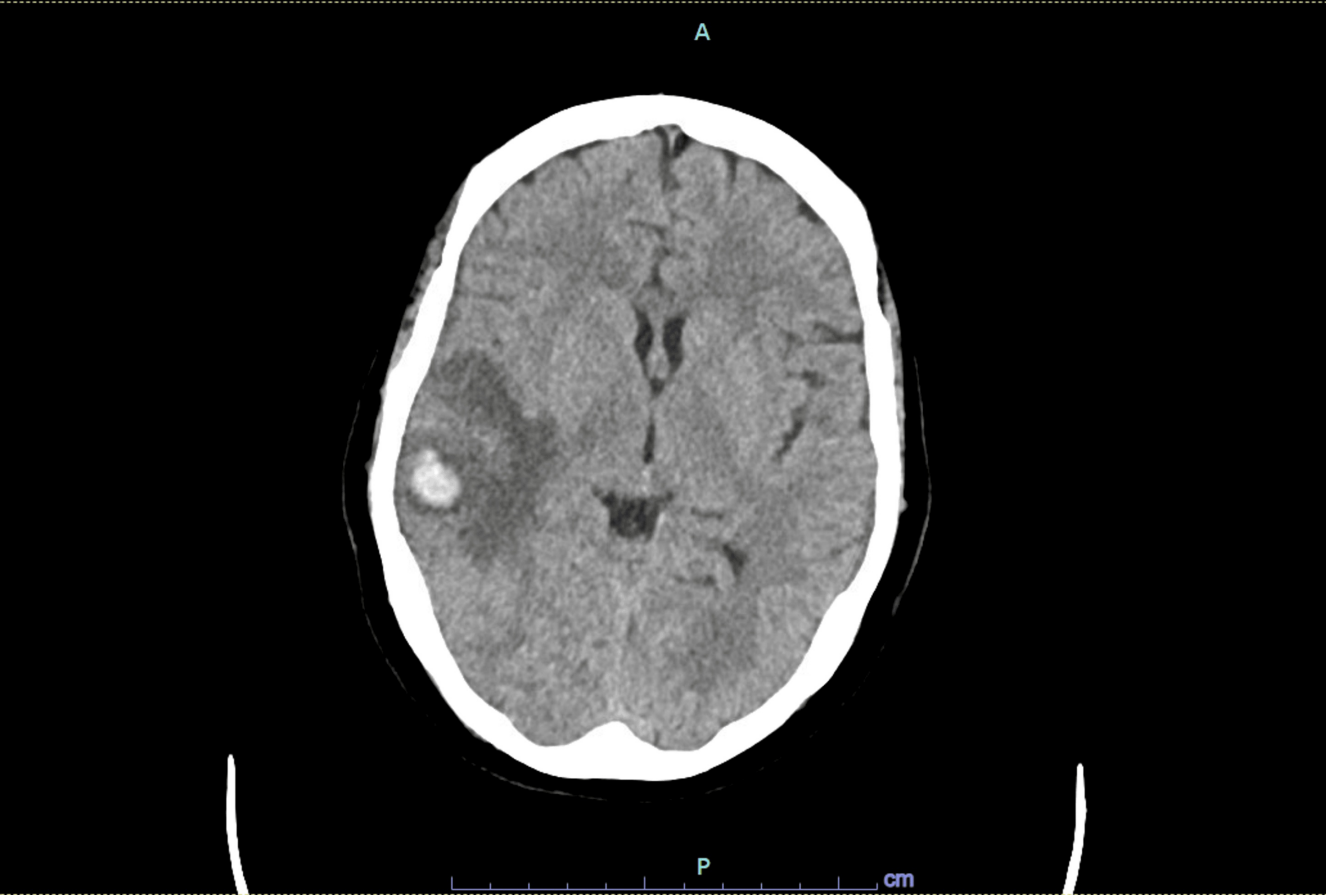
CT head without contrast showing acute right temporal intraparenchymal hematoma (axial view)

**Figure 3:**
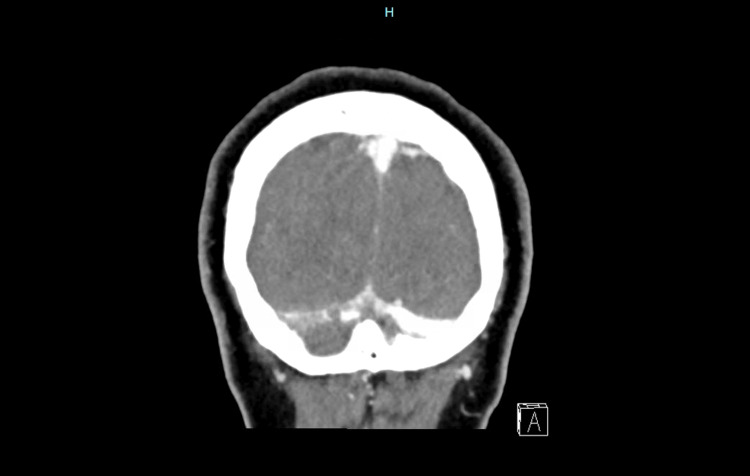
CT venogram showing right transverse sinus thrombosis

Serum chemistries and coagulation profile were unremarkable. Complete blood count revealed a hemoglobin of 14 g/dL, a white blood cell count of 12,600/µL, and a platelet count of 494,000/µL, consistent with mild hemoconcentration. Initial CT head showed an acute right temporal intraparenchymal hematoma measuring 12.8 x 15.8 x 7.7 mm, with moderate surrounding edema (Figures 1, 2). CT venogram revealed right transverse sinus thrombosis (Figure 3).

Intravenous fluid infusion was started in addition to low molecular weight heparin (LMWH) therapy. Serial CT scans and periodic neurologic assessments remained stable. Serum homocysteine level and repeat complete blood count values were within normal limits. The patient was transitioned to apixaban and it was continued for six months. Quarterly follow-up visits did not reveal any concerning clinical findings. Comprehensive thrombophilia work-up was undertaken after the completion of anticoagulation therapy. The patient tested negative for antithrombin, protein C and S deficiency, anti-phospholipid antibodies, and factor V Leiden and prothrombin G20210A mutations. The patient was up-to-date with current malignancy screening protocols. At her one-year follow-up visit, the patient remained completely asymptomatic with no discernible objective evidence of any hereditary or acquired thrombophilias. 

## Discussion

Up to 90% of patients with CSVT present with headaches. Our patient also complained of a debilitating headache on presentation. The majority of patients (80-90%) with CVST make an uneventful recovery and reach their pre-disease state of functional independence after successful treatment. Few patients, however, experience sequelae such as persistent headaches, mood disturbances, cognitive dysfunction, or fatigue that can impede the achievement of optimal functional status [4]. Patients with seizures, coma, focal neurological deficits, juxtacortical hemorrhages, or superior sagittal sinus thrombosis on presentation can develop epilepsy (~10%) as a long-term complication [5]. CVST can also result in the formation of dural arteriovenous fistulas, and vice versa. 

Computed tomography (CT) or magnetic resonance (MR) imaging can reveal venous thrombosis, infarction, cerebral edema, or hemorrhagic transformation. Hypodensities with dural venous sinuses can also be evident. Juxtacortical hemorrhages are seen in up to 40% of cases. A recent meta-analysis analyzing data from 2800 patients determined that CT imaging had 79% sensitivity and 90% specificity in diagnosing CVST, whereas MRI had 82% sensitivity and 92% specificity [6]. CT and MR venography are deemed optimal diagnostic modalities for CVST. However, CT venography (CTV) has a lower sensitivity for cortical vein thrombosis. MR venography (MRV) can be performed with or without contrast-enhanced (CE) techniques. CE-MRV affords superior characterization between hypoplastic sinuses (flow gaps) and low-flow states despite having a comparable sensitivity and specificity to CTV. Cortical venous thrombosis is best assessed by CE techniques and susceptibility-weighted imaging or gradient-recalled echo [7]. We had initially utilized CTV to expeditiously assess for CVST in our patient. Since CTV clearly revealed the presence of transverse sinus thrombosis, we decided to forgo MRV. Nevertheless, MRV should be pursued in cases with negative CTV imaging and ongoing suspicion for CVST. 

Initial management of CVST involves the use of LMWH or unfractionated heparin (UFH) in patients with or without hemorrhagic venous transformation. LMWH is preferred due to a feasible administration schedule, predictable therapeutic effect, and lower incidence of thrombocytopenia. Anticoagulation therapy is warranted for 3 to 12 months in cases with transient risk factors, and indefinitely for patients with chronic major risk factors or an underlying thrombophilic disorder [8]. Outpatient thrombophilia work-up for our patient was negative for antithrombin, protein C and protein S deficiency, factor V Leiden and prothrombin gene mutation, and anti-phospholipid antibodies (APLA). 

The incidence of venous thromboembolism (VTE) increases after an episode of CVST, ranging between 1-4% annually. The CVST recurrence rate is less than 2% per year but could be higher in patients with thrombophilic disorders. VTE and CVST recurrence rates in the ACTION-CVT study were 6.4% and 2.5%, respectively [9]. A Norwegian study with a sample size of 654 found that younger patients with CVST had an increased risk of recurrent VTE compared with age- and gender-matched control subjects. Older patients (age ≥ 55 yrs), on the other hand, had a higher risk of mortality, major bleeding events, and ischemic stroke at 10 years [10]. 

Recent evidence has shown that direct-acting oral anticoagulants (DOACs) are a reasonable alternative when compared with warfarin due to comparable safety and efficacy estimates. Our patient completed a 6-month course of apixaban therapy. She is currently symptom-free and has resumed her baseline functional status. She has not had any thromboembolic events for two years since the resolution of her first CVST episode. 

RE-SPECT CVT was a randomized trial that compared warfarin with dabigatran amongst 120 cases with CVST who had received 5-15 days of parenteral anticoagulation [11]. It excluded patients with malignancies, trauma, pregnancy, and central nervous system infections. Recurrent VTE was not reported in either group. The SECRET trial assessed the efficacy of rivaroxaban in comparison to warfarin or LMWH in patients who had not received lead-in parenteral anticoagulation [12]. At six-month follow-up, one event of recurrent CVST, one event of symptomatic intracranial hemorrhage, and two events of non-major bleeding were reported in the rivaroxaban group. No recurrent CVST or bleeding events were noted in the control arm. ACTION-CVT compared outcomes with warfarin versus DOAC treatment in 845 CVST cases (excluding patients with malignancy, pregnancy, and APLA syndrome). Rates of recurrent VTE were not statistically different amongst the two groups [2]. A 65% reduction in the risk of major bleeding episodes (primarily driven by a lower rate of ICH) was noted in the DOAC group. A large meta-analysis also found comparable rates of recurrent VTE and major bleeding events amongst DOAC and warfarin subgroups [13]. 

No consensus has been achieved on the optimal timing of DOAC initiation and the need for lead-in heparin therapy [11]. Also, select subgroups are not suitable candidates for DOAC treatment. For example, only LMWH is recommended for pregnant and breastfeeding patients. For cancer patients, LMWH is the agent of choice but DOACs are considered non-inferior to LMWH. Also, warfarin is indicated in APLA syndrome due to the high incidence of DOAC failure [14].

Evidence on the efficacy of endovascular treatment (EVT) has failed to show any mortality benefit. The TO-ACT trial showed that EVT did not result in statistically significant clinical benefit compared to standard anticoagulation protocol [15]. Some meta-analyses have failed to show any clinical benefit of EVT, and rather, have found higher mortality rates in patients receiving EVT [16, 17]. Currently, EVT is reserved only for patients who continue to show clinical deterioration despite optimal anticoagulation (AC) therapy, or those who have absolute contraindications to AC [18]. Decompressive craniectomy is indicated in patients with impending herniation. Studies have shown that craniectomy within 48 hours of presentation improves functional outcomes and provides mortality benefits [19]. 

## Conclusions

This case highlights the development of CVST due to severe dehydration. Given the vague symptomatology, it is imperative to evaluate for CVST using CT or MR venography in suspected cases. Treatment is anticoagulation in cases with or without hemorrhagic venous transformation. Timely initiation of anticoagulation resulted in a favorable outcome in our case. It is important to rule out an underlying malignancy and other thrombophilias after resolution of the acute episode.
